# Address

**Published:** 1874-02

**Authors:** E. J. Waye


### ADDRESS.

BY E. J. WAYE.

*Before the Ohio State Dental Society.*

Mr. President and Gentlemen : I have selected the second on the list as the subject for discussion in this paper, viz: "How much is the success of filling teeth due to mere manipulation ?"

To the question as stated there can be but one reply : the whole. For filling teeth is merely a manipulative performance, and unless we include in the term filling the treatment which so often precedes it, and which is no less essential to the success of the operation than the filling itself, the question admits of but one reply.

Had the question read, "How much of success in *saving* teeth depends upon mere manipulation ?"—there would then be another side to it. The discussion of which might possibly result in benefit to the profession. If the Society will permit, I will assume that such was the meaning of the question, and upon that ground proceed briefly to discuss it.

In the saving of teeth, much, very much, depends on mere manipulative skill. True, this must be coupled with knowledge to direct it ; for, however finished and elegant the filling, it will fail to save the tooth if the disease remain. And the skill which is *merely* manipulative is in no sense adequate to diagnose and treat disease.

This requires a thorough knowledge of the anatomy, physiology and pathology of the teeth and their connections, together with the diseases to which they are incident, and the treatment necessary to restore them to health. And this theoretical knowledge must in every case precede the manipulation; otherwise all that skill and nicety which, directed by knowledge, would insure success, might, and in all probability would be, *wasted* skill, time and reputation. It is this knowledge which is the foundation for all success in dentistry. Without it, manipulation, however finished and delicate, is mere groping in the dark, with results always doubtful, and failure the rule. The sooner this becomes a settled axiom, the sooner, it is hoped, will our young aspirants for professional honors manifest an earnestness in its acquisition equal at least to their former indifference, and the coming dentist, laying this sure foundation, be built up in such symmetry and perfection as shall make him the fit associate and equal of the best minds in every specialty of medicine and surgery.

Within the past few years, it has been a favorite ambition with our young men to excel in operative dentistry. I use the term not in the comprehensive sense in which it is now understood by the intelligent and educated in the profession, but rather as defined by Prof. C. M. Wright, whose brilliant coruscations of wit and irony no longer scintillate in the pages of the REGISTER, whose fame is now trans-Atlantic, and whose loss as a pyrotechnic writer upon operative dentistry and dentists we all so deeply deplore. Here is his definition—"What is operative dentistry? It is the surgical operation of cutting away decomposed and decomposing dentine and enamel of teeth—cutting the cavities into good shape for retaining foreign substances, and then stuffing, stopping, plugging or filling the cavity with gold, tin, amalgam or oxy-chloride of zinc."

Farther on, he characterizes operative dentists under this definition, but with superior manipulative skill, as "mouth jewelers," a not inappropriate appellation. Now this kind of operative dentistry is what is understood by a "*mere*" manipulator, and he is apt to be curiously vain of his specialty; looks down upon mechanical dentistry as something too common for his refined ideas. "Does none of it;" "preserves the natural organs," and feels several planes above the mechanical dentist, however skilful and artistic his work; is somewhat apt to come down rather heavy on men whose knowledge very far exceeds his own, but whose skill as "mouth jewelers" is less, and pointing to his own elegant fillings he asks, "Can so and so with all his knowledge do such work as that?" Whenever any question relating to physiology, pathology or the treatment of disease is under discussion, he either goes out or with a suppressed yawn inquires of the man next him "When will these old fogies get through?" Is always glad, when "How to fill teeth" or "Dental instruments" comes up, feeling then quite at home. And usually has some new plugger, (all his own,) with a new point, or a new shape, with a lump on the handle, in a new place, or a novel crook for reaching posterior cavities.—or an ovoid, spheroid, convex surface, unlike anything before seen, "his own particular invention," and yet strangely similar to what may be found in any dental depot of five years standing laid away in some drawer or on a back shelf to "blush unseen" till some tyro who wants "all the modern shapes" comes in, when it and himself are sold together—to the intense gratification of both depot man and buyer at the time, though how long that will last we will not attempt to predict.

Your operative dentist goes in strongly for clinics at all times. Says "If you want to teach a man anything, *show* him how to do it," and it will usually be found that, quite by accident you might say, he has his instruments with him, and is not unwilling to enlighten the uninitiated in all the mysteries of coffer-dam, heavy foil, heavy or light steel or lead mallets, matrices, clamps, *et cetera*, wholly and purely for the good of said uninitiated, and *not* that his own name may appear as the clinical demonstrator, with such flattering remarks as may be added by a disinterested reporter. But why continue? You know how it is yourselves, gentlemen, and you can but be satisfied that mere operative or manipulative skill, while it is a grand essential in the make up of the accomplished dentist, is in and of itself of little value, and that the making it the one all important study and aim tends to narrow and dwarf the best impulses and aspirations of the mind, to such a degree as to produce only a dextrous and exquisite manipulator; vain of his own skill, jealous of others, and ignorant of the higher demands of the profession upon him; whereby the community, his brethren and himself are all made to suffer for, instead of being what both community and the profession have a right to expect, viz : a dentist in the best and highest signification of the term, possessing a knowledge of the organs and their structure, and their relation to the surrounding parts, their diseases and the proper treatment to restore them to health ; together with such manipulative ability as will secure permanence and beauty to the operation, (and nothing less than this constitutes a dentist,) he will have become a mere artisan, doing beautiful and finished work upon the teeth, with little more knowledge of the delicate and sensitive organs under his hands than has the artisan who, in gold, silver, ivory or cameo, works out those exquisite designs which are the admiration of all beholders. But while this is true of mere manipulation, is there nothing to be said of mere knowledge ? Admitting, that knowledge is the foundation of all success in dentistry, if we go no farther, what becomes of the superstructure ? Of what use is the foundation alone ? It is almost as useless as would be the superstructure without it.

Of what use is it to the surgeon that his knowledge should enable him to locate with unerring certainty each nerve and artery, bone and ligament, if to this be not added an equally thorough acquaintance with surgical instruments and the manner of using them, together with the deft and cunning fingers, the accurate and quick eye, and the steady nerve ? *Mere* knowledge could not perform the operation, and if undertaken without its all essential auxillary, manipulative skill, what should we expect but a disastrous result ?

So in our own profession, of what avail is the knowledge of disease, and the treatment which heals, if the same hand shall fail in that manipulative skill which gives to the restored organ beauty and permanency ?

Gentlemen, is it not plain that knowledge and manipulation to be effective must be united ? that separated, they never have, nor can, accomplish the best results for either dentist or patient ?

If such be your conclusion, and I cannot doubt it, nothing further in the way of argument need be added, and I close with an anecdote whereby to "point the moral:"

A young minister of my acquaintance, who has since made his mark in the religious world, speaking of the qualities which command success, said to me : "Doctor, so far as success in life is concerned, mere merit is of little value ; humbug and assur-are no better : but unite them, and you are irresistible. Assurance you must have to get yourself before the people, and, when there, real merit to stand upon."

So as regards success in our profession, *mere* knowledge is of little use ; *mere* manipulative ability is no better : "Unite them and you are irresistible."

